# What's on the Inside Counts: A Grounded Account of Concept Acquisition and Development

**DOI:** 10.3389/fpsyg.2016.00402

**Published:** 2016-03-23

**Authors:** Serge Thill, Katherine E. Twomey

**Affiliations:** ^1^Interaction Lab, School of Informatics, University of SkövdeSkövde, Sweden; ^2^Department of Psychology, Lancaster UniversityLancaster, UK

**Keywords:** concept grounding, embodiment, developmental linguistics, age of acquisition, SPA

## Abstract

Understanding the factors which affect the age of acquisition (AoA) of words and concepts is fundamental to understanding cognitive development more broadly. Traditionally, studies of AoA have taken two approaches, either exploring the effect of linguistic variables such as input frequency (e.g., Naigles and Hoff-Ginsberg, 1998) or the semantics of the underlying concept, such as concreteness or imageability (e.g., Bird et al., 2001). Embodied theories of cognition, meanwhile, assume that concepts, even relatively abstract ones, can be grounded in the embodied experience. While the focus of such discussions has been mainly on grounding in external modalities, more recently some have argued for the importance of interoceptive features, or grounding in complex modalities such as social interaction. In this paper, we argue for the integration and extension of these two strands of research. We demonstrate that the psycholinguistic factors traditionally considered to determine AoA are far from sufficient to account for the variability observed in AoA data. Given this gap, we propose *groundability* as a new conceptual tool that can measure the degree to which concepts are grounded both in external and, critically, internal modalities. We then present a mechanistic theory of conceptual representation that can account for groundability in addition to the existing variables argued to influence concept acquisition in both the developmental and embodied cognition literatures, and discuss its implications for future work in concept and cognitive development.

## 1. Introduction

Within representationalist theories of embodied cognition, the symbol grounding problem has traditionally received much attention. The reason for the focus can be understood from a historical perspective: as Chemero (2009) notes, these theories developed primarily as a reaction to purely computationalist views of cognition^1^. One of the main criticisms leveled at such views was that they assume amodal symbols which are meaningless to the system itself—whatever meaning the symbols might carry was attributed by external observers. How such symbols could acquire meaning that is intrinsic to the system became known as the symbol grounding problem (Harnad, 1990), and the central claim to the solution in embodied terms is that the meaning is acquired through sensorimotor interaction with the world.

This has led to at least two major research strands. On the more experimental end of the spectrum, much work has focused on detailing the involvement of sensorimotor areas of the brain in, for instance, language processing (see Chersi et al., 2010, for a review). Although such involvement is often taken as evidence for a grounded or embodied understanding of concepts, it is worth pointing out that this is not uncontroversial: Mahon and Caramazza (2008), for instance argue, that the evidence is not sufficient to invalidate disembodied hypotheses.

On the computational end of the spectrum, researchers are interested in creating models of symbol grounding. Eliasmith (2013), for example, details a “semantic pointer architecture,” which provides a computational implementation of many aspects of Barsalou's perceptual symbol system (Barsalou, 1999). Other efforts consider robotic implementations of such models (see for instance, Stramandinoli et al., 2012, or for a review, Coradeschi et al. 2013).

A particularly interesting aspect of research across the entire spectrum concerns the putative grounding of abstract concepts—that is, concepts which do not have a directly perceivable sensorimotor target (see, for instance Dove, 2011; Thill et al., 2014, for recent reviews and discussions). While it is relatively straightforward to propose accounts of sensorimotor grounding of concrete concepts—which do have an observable sensorimotor target in the external world—it is less clear how, if at all, abstract concepts should relate to embodied experience. Mahon and Caramazza (2008) give the example of the concept “beautiful,” for which they claim that there is no corresponding *consistent* sensory or motor information (their emphasis).

An early attempt at explanation is given by the conceptual metaphor theory (Lakoff and Johnson, 1980), which postulates that metaphors and analogical reasoning (e.g., an argument is like war; happiness is up) mediate grounding of abstract concepts in direct sensorimotor experience. However, Dove (2011) points out that the required cognitive mechanisms, such the ability to construct such analogies and metaphors, are not likely to develop until relatively late. He further argues that linguistic representations are dis-embodied (the specific term he coined, and distinct from disembodied) in the sense that they do not acquire semantic content from embodiment, even though they may remain dynamic, multimodal and grounded in linguistic experience. Zwaan (2015) also argues that abstract concepts “acquire a specific sensorimotor instantiation in a discourse context” while being only weakly associated with sensorimotor representations. Similarly, Barsalou et al. (2008) previously proposed the Language And Situated Simulation (LASS) theory, arguing that both linguistic forms and situated simulations are used to represent concepts, including abstract ones.

Other theories imply that the grounding of more abstract concepts can take place in modalities beyond the five senses in the strict sense. The Words As Tools theory (WAT; Borghi and Binkofski, 2014) sees words as social tools, whose use is a “type of experience” (Borghi and Cimatti, 2012, p. 22), which provides a potential way of grounding abstract concepts in a type of social modality. Similarly, Thill et al. (2014) argue that one should not restrict the embodied experience to the “outside” in a theory of concept grounding while Wellsby and Pexman (2014a) note that the focus so far has been more on interaction with the external world and less on “sensing bodies” (their term). This is also true for theories that try to link abstract concepts to embodiment, for instance by grounding them in the sensorimotor representations activated across different linguistic contexts (Barsalou and Wiemer-Hastings, 2005; Zwaan, 2015). As others have noted, the human embodied experience is actually very rich and involves many internal processes (see Stapleton, 2011, 2013, for a thorough review and discussion), including homeostatic and affective mechanisms (e.g., Ziemke and Lowe, 2009; Damasio, 2010) which may directly ground concepts that are considered abstract. As noted by Stapleton (2013), the *internal body* may^2^ matter to cognition. Of the aspects that comprise this internal body, affect and emotion have received the most attention in discussions of concept grounding so far. Glenberg and Gallese (2012), for instance, propose an account of language acquisition that includes emotional systems as a providing means for grounding in addition to perception and action. Similarly, Kousta et al. (2011) argue that abstract words tend to be more emotionally valenced than concrete ones, and that *emotional content* might be an important factor in the representation and processing of abstract words in particular. Newcombe et al. (2012) showed a correspondence between emotional experience and speed (and accuracy) of classification of abstract—but not concrete—words, and argue that abstract concepts may be grounded in emotional features that remain stable across different contexts (see also Siakaluk et al., 2014, for a follow-up). The concept of “beautiful,” although having no consistent *external* sensorimotor experience, may thus relate to direct internal experience.

Research into concept grounding tends to focus on adult language and cognition. There are, however, good reasons to approach the topic from a developmental perspective (Kontra et al., 2012). Most immediately, any mechanistic account of concept grounding makes the direct prediction that whatever mechanism is proposed has developed by the time that humans use that concept—recall, for example, Dove's (2011) concern regarding the use of metaphors previously mentioned. Second, bodily and cognitive development may be a crucial component for explanatory accounts of cognitive mechanisms: after all, humans acquire concepts during a period of dramatic change.

Concept grounding depends, by definition, on the sensorimotor experience that is meant to provide this grounding. The importance of this embodied input has been accepted since Piaget's classic work on the sensorimotor roots of cognitive development (Piaget, 1952). More recently, however, new technology has provided striking novel insights into the infant's embodied experience: that is, what infants experience is substantially different from what adults experience. As the body changes—e.g., arms grow longer, walking commences—so too do important characteristics of the body-mediated information available for concept grounding. Studies using head-mounted eye trackers demonstrate, for example, that the content of the infant's visual field is qualitatively and quantitatively different from that of the adult, because infants' shorter arms lead them to hold objects close to their faces (Smith et al., 2011). The precise nature of the body (e.g., walking vs. crawling, height) is clearly crucial in shaping this experience (Kretch et al., 2014); yet it is also often ignored in the embodied cognitive science literature. For instance, Ziemke (2003) points out that “many discussions/notions of em*bodi*ed cognition actually pay relatively little attention to the nature and the role of the body involved (if at all)” (p. 1306, emphasis in text) and Borghi et al. (2013) similarly argues that “many versions of the [embodied-grounded] view are too brainbound” (p. 2).

The developmental psychology literature also features a substantial body of work concerned with human concept and word acquisition. This work is highly relevant to the concept grounding discussion. In particular, it illustrates how change over time in the conceptual system reflects change over time in the physical system. For instance, conceptual structure changes radically across development (Quinn and Eimas, 1997; Mandler, 2000): infants as young as 3 months form perceptually-based categories (Quinn et al., 1993), but begin to show evidence of more abstract representations by around 12 months (Mandler and Bauer, 1988), and make conceptually-based category judgements by 4 years (Keil, 1989). Importantly, early perceptual/conceptual structure and language acquisition are intimately linked. For example, by drawing attention to invariant, category-relevant features, perceptual variability in the objects children see supports category formation and subsequent word learning (e.g., Vlach et al., 2008; Twomey et al., 2014; Goldenberg and Johnson, 2015). Relatedly, English-learning children generalize category labels to new same-shape items, but only if those items are solid rather than non-solid (Samuelson and Horst, 2007). Further, variation in the physical position of the body can disrupt word learning (Samuelson et al., 2011; Morse et al., 2015). Thus, evidence from multiple modalities indicates that the perceptually grounded nature of early concrete concepts interacts with children's ability to learn words. Indeed, the interaction between perceptual grounding and early language has been investigated. For example, in a word naming study which included school-age children, Wellsby and Pexman (2014b) demonstrated that the extent to which the referents of words are easy to physically interact with (as rated by adults) affected 8- to 9-year old children's written word processing. Specifically, children's naming latencies were shorter for words with high body-object-interaction (BOI) ratings. The authors argued that high-BOI words have richer semantic representations than low-BOI words, leading to greater activation in the semantic system, which in turn facilitates word recognition. Taken together with the adult literature, the developmental embodied cognition approach makes the prediction that the sensorimotor experience associated with a concept should affect how easy it is to acquire that concept.

Recent psycholinguistic studies have focused on the age of acquisition (AoA) of words as a marker of concept learning, and demonstrate that the semantic features of concepts themselves affect the age at which their labels are learned. For example, McDonough et al. (2011) examined the effect of a word's imageability (the extent to which a word generates a mental image, Paivio et al., 1968) and class (e.g., noun, verb) on AoA. As well as predicting AoA, imageability accounted for variation that word class did not, indicating an independent role of perceptual features in the acquisition of early abstract concepts (for crosslinguistic evidence, see Ma et al., 2009). Closely related to imageability is concreteness, or the extent to which a concept is perceptible (Brysbaert et al., 2014). Bird et al. (2001) showed that imageability and concreteness predicted AoA for children's early-produced nouns (see also Barca et al., 2002; Smolík, 2014). In a study in which Dutch adults rated words for emotional valence, arousal, power and AoA, valence was negatively correlated with AoA such that more positive words were acquired earlier (Moors et al., 2013). In addition, linguistic phenomena also affect AoA, including—but not limited to—iconicity (Perry et al., 2015), and in particular, input frequency (Naigles and Hoff-Ginsberg, 1998; Barca et al., 2002; Storkel, 2004; Goodman et al., 2008; Ambridge et al., 2015; Roy et al., 2015). Whether sensorimotor experience predicts AoA, however, remains to be tested.

In the following section we bring together in a single analysis variables that have been shown to affect AoA, specifically, frequency, imageability and valence. Our goal is not to provide an exhaustive account of conceptual and linguistic influences on AoA; indeed, for many of these variables insufficient data are available for a reliable analysis. However, to our knowledge this is the first study to bring together these variables in analysing the reliable measure of AoA provided by the widely-used MacArthur-Bates Communicative Development Inventory vocabulary norms (Fenson et al., 1993). We demonstrate that, when taken together, these variables explain only a minority of the variance, highlighting the importance of identifying and testing new factors. In a second analysis we test our hypothesis that sensorimotor grounding is important to AoA, by adding a measure of body-object interaction. We argue that while existing measures take into account conceptual and linguistic effects on AoA, embodied characteristics of concepts may be an important missing piece of the puzzle.

## 2. Methods

To explore the effect of conceptual features on AoA we obtained AoA, frequency, imageability and valence ratings from a range of open access sources. Data used in the analyses are provided in Supplementary Materials and Pearson correlations between variables are presented in Table 1.

**Table 1:** **Pearson correlations between regression predictors**.

	**BOI**	**Imageability**	**Frequency**
Imageability	0.44^***^		
Frequency	0.18	−0.45^***^	
Valence	−0.21^*^	0.23^***^	0.22^***^

* *p < 0.05*,

*** *p < 0.001*.

### 2.1. Age of acquisition

Our goal was to explore the extent to which previously identified variables predict the AoA of words commonly learned by human infants. We took our target words from the MacArthur Bates Communicative Development Inventory (MCDI; Fenson et al., 1993). The MCDI is a well-established, normed and validated list of 680 words that infants and toddlers learn to understand and produce up to 30 months of age, and is widely used in developmental research. We defined AoA as the month in which 50% or more of 1142 infants in the MCDI sample produced a given word. AoA in months ranged from 12 (e.g., *mommy*) to 30+ (e.g., *pretend*). AoAs listed as 30+ months were coded as 31 months for the purposes of the current analysis.

### 2.2. Frequency

Children's language environment has been repeatedly shown to influence their language acquisition (for a review, see Ambridge et al., 2015). We therefore generated our frequency data from real child-directed input, which is representative of the language children hear, rather than relying on corpora of non-child-directed spoken or written speech. CHILDES (MacWhinney, 2000) is a large, open-access online database of transcribed, naturalistic conversations between adults and children. We searched all Northern American corpora for each word in the MCDI, with the exception of some sound effects and routines (e.g., *woof*, *patty cake*). Only mothers' utterances were queried, providing an index of children's input. This resulted in frequency ratings for 638 words with frequencies ranging from 0 (*cat*) to 128124 (*you*) tokens (*M* = 2848.82).

### 2.3. Imageability and concreteness

For each MCDI word for which we obtained frequency data we extracted imageability and concreteness ratings from the MRC Psycholinguistic Database (Coltheart, 1981; Wilson, 1988). The database is a large, open-access collection of 26 psycholinguistic variables for up to 150,000 words (although not all words have data for all variables) aggregated from existing studies^3^. Because imageability and concreteness were very highly correlated (*r* = 0.91, *p* < 0.0001), in line with Ma et al. (2009) and McDonough et al. (2011), we used imageability as a predictor variable in the following analyses. Imageability scores ranged from 195 (low) to 667 (high; *M* = 495.58).

### 2.4. Valence

Valence ratings for each word were taken from the 2010 version of the Affective Norms for English Words dataset (ANEW; Bradley and Lang, 2010). This version of ANEW consists of adult ratings of 2476 words for pleasure (i.e., valence), arousal and dominance. Scores ranged from 1.61 (happy) to 8.72 (unhappy; *M* = 5.92).

### 2.5. Body-object interaction

To explore our hypothesis that sensorimotor grounding may be important for concept acquisition, we took measures of body-object interaction (*BOI*) from Tillotson et al. (2008) and Bennett et al. (2011), in which adults were asked to rate the extent to which they could easily interact with a named item. Scores ranged from 1.27 (*first*; low interactivity) to 6.43 (*doll*; high interactivity; *M* = 4.68). Specifically, our assumption is that the experience of interacting with concepts that rate highly is more multi-modal than that of interacting with low-ranking concepts (if such an experience exists at all), so BOI might serve as a proxy to rank concepts by how much they are defined by an external sensorimotor experience.

## 3. Results

### 3.1. The effect of conceptual features on AoA

To explore the effect of conceptual features on AoA, we first created a *conceptual features model*. AoA for the 398 words with ratings for every variable was submitted to a linear regression with frequency (log transformed), imageability (mean centred) and valence (mean centred) as fixed effects. Because high frequency function words have little or no semantic content, while rarer nouns have rich semantics, we anticipated that frequency and imageability would interact, so included a frequency-by-imageability interaction term (cf. Roy et al., 2015).

**Figure 1:**
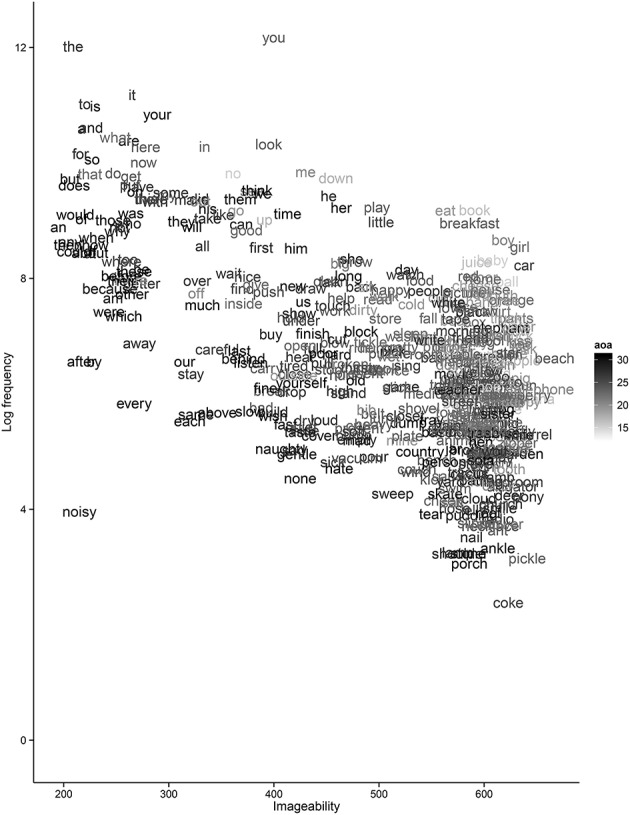
**AoA of early concepts plotted by log frequency and imageability**. Darker text indicates later AOI.

Results are presented in Table 2. The principal result is that the interaction between frequency and imageability predicts AoA, extending the findings of McDonough et al. (2011) and Ma et al. (2009), who each found correlations between CDI AoA and imageability ratings. As illustrated in Figure 1, although late-acquired words tend to be lower frequency, function words (e.g., *an, the, to*) have low imageability and are acquired late despite being high frequency. In contrast, high-imageability words for the things infants encounter in their everyday environment (e.g., *puppy*) are acquired early despite occurring infrequently. In addition to the interaction between imageability and frequency, main effects of these two variables confirmed that as imageability increased, AoA decreased (see also Ma et al., 2009; McDonough et al., 2011), and in line with Roy et al. (2015), as frequency increased, AoA decreased. Interestingly, in contrast with existing studies (e.g., Bird et al., 2001; Moors et al., 2013), valence did not predict AoA; however the adult ratings we used may not capture the effect of a word's valence on young children. More broadly, the differences between our results and existing studies may stem from some important methodological differences: while the majority of work uses adult ratings of word AoA and frequency measures taken from corpora of adult-directed language, we use parental measures of their own children's language and frequencies taken from child-directed speech (*cf*. McDonough et al., 2011). This contrast highlights the need for child-centric ratings of such predictors, and illustrates the importance of taking seriously the real input to infants when investigating developmental phenomena (Smith et al., 2011).

**Table 2:** **Conceptual features model parameters and significance tests (***N*** = 239)**.

	**β**	***t***	***p***	***F***	***df***	***p***
Overall model				37.73	(4, 234)	< 0.0001^***^
Log frequency	−1.48	−6.74	< 0.0001^***^			
Imageability	−0.022	−7.33	< 0.0001^***^			
Valence	0.055	0.34	0.74			
Log frequency × imageability	−0.0065	−3.18	0.0017^**^			
*R*^2^	0.39					
Adjusted *R*^2^	0.38					

** *p < 0.01*,

*** *p < 0.001*.

The goal of this analysis was to illustrate that even well-tested predictors are unable to fully explain AoA. As expected, this model accounted for less than half of the variance (adjusted *R*^2^ = 0.38), leaving substantial scope for the influence of other factors on early concept acquisition. As noted above, our analysis focuses on variables which have repeatedly been shown to influence AoA, and ignores those for which no data are available. Thus, we do not claim that it is an exhaustive model of the factors affecting concept AoA. We do, however, argue that the variance unaccounted for is not simply random variation, but rather the result of linguistic and concept-internal variables not typically included in analyses of AoA. In particular, this leaves open the possibility that embodied aspects of concepts may contribute to the ease with which they are acquired.

### 3.2. The effect of a sensorimotor grounding on AoA

To explore whether the extent of sensorimotor grounding might play a role in concept acquisition (as discussed in Section 2.5), we added a measure of body-object interaction as a predictor in the conceptual features model to create a *BOI model*. Because fewer of our target words had ratings for this variable, the final dataset for this analysis consisted of complete ratings for 151 words.

As illustrated in Table 3, when the additional BOI term is included, the frequency-by-imageability interaction and main effect of imageability predict AoA, while the main effect of frequency does not. Critically, in line with our predictions, BOI does predict AoA, such that as words are rated as more difficult to interact with, AoA increases. Importantly, this model also explained a greater proportion of the variance in AoA, with an increase in adjusted R-squared from 0.38 to 0.40. To compare the fit of our two models, we first refit the conceptual features model to the smaller dataset; this resulted in a similar pattern of results (see Table 4). Including the BOI term resulted in a reduction in AIC from 788.43 to 770.80. Taken together with the increase in adjusted R-squared, this confirms that the BOI model fits the data better, explaining more variance than the conceptual features model and supporting our claim that the extent to which concepts are grounded in the body affects AoA.

**Table 3:** **BOI model parameters and significance tests (***N*** = 151)**.

	**β**	***t***	***p***	***F***	***df***	***p***
Overall model				21.32	(5, 145)	< 0.001^***^
Log frequency	−0.93	−1.78	0.078			
Imageability	−0.013	−2.22	0.028^*^			
Valence	−0.19	−0.76	0.45			
Body-object interaction	−0.88	−4.49	< 0.001^***^			
Log frequency × imageability	−0.010	−1.99	0.049^*^			
*R*^2^	0.42					
Adjusted *R*^2^	0.40					

* *p < 0.05*,

*** *p < 0.001*.

**Table 4:** **Conceptual features model parameters and significance tests fit to dataset used for BOI model (*N* = 151)**.

	**β**	***t***	***p***	***F***	***df***	***p***
Overall model				19.11	(4, 146)	< 0.001^***^
Log frequency	−0.59	−1.07	0.28			
Imageability	−0.022	−3.59	< 0.001^***^			
Valence	0.037	0.27	0.14			
Log frequency × imageability	−0.014	−2.62	0.0099^**^			
*R*^2^	0.39					
Adjusted *R*^2^	0.38					

** *p < 0.01*,

*** *p < 0.001*.

Although including BOI improved the fit of the model, it nonetheless again left a majority of the variance unaccounted for—as expected, given that it did not include linguistic effects on AoA, for example iconicity (Perry et al., 2015), ease of pronunciation (Jorm, 1991) and contextual diversity (Hills et al., 2009), and the fact that these ratings came from adults. Thus, it is, for example, possible that using child ratings of BOI could improve the model fit further. What drives concept AoA is far from being fully understood; however the above analyses strongly suggest that grounding in sensorimotor experience could be a critical piece in this puzzle.

## 4. What are concepts made of?

To summarize the results, we first showed that semantic features and linguistic phenomena such as frequency are not sufficient to explain AoA data. Our main hypothesis is that this is because such features do not take into account grounding in a rich or proper sensorimotor experience. We then demonstrated that including predictors related to such a grounding improve on the initial results.

There is clearly much work to be done to validate the hypothesis further. First and foremost, there are currently no major corpora of data that relate to relevant measures other than BOI as used above. Second, the measure of BOI used above takes no account of interoceptive aspects of the sensorimotor experience, which, as noted, are likely to play a part in conceptual structure. How to tap into such interoceptive aspects is not trivial. Although valence ratings may seem like a good starting point (since valence itself is part of the internal sensory experience), they do not provide a measure of how diverse (or multi-modal) the internal sensory experience associated with a concept is^4^. Instead, they quantify the strength of one aspect (which is clearly relevant, as argued for instance by Kousta et al., 2011, but not necessarily sufficient since there are other internal modalities as discussed, for example, by Stapleton 2011). Together with the limitations of BOI mentioned before, there is therefore still a need for designing new types of measures that address both internal and external sensorimotor experience more explicitly.

The purpose of the remainder of this paper is therefore to outline a mechanism of concept learning which explicitly takes into account embodied features beyond simple sensorimotor interaction (for instance, interoceptive features) whilst incorporating the variables which have been repeatedly shown to affect AoA, and by extension, conceptual development and structure. In doing so, we will generate testable predictions for future work and lay the groundwork for future research into novel measures that can validate our hypothesis.

To provide this characterization, we cast our discussion in terms of a cognitive architecture since these necessarily formally specify the mechanisms underlying concept use. Specifically, we base our discussion on the semantic pointer architecture (SPA, see Eliasmith, 2013). It would of course be equally possible to formulate these ideas in frameworks other than SPA; the Neural Blackboard Architecture framework (van der Velde and de Kamps, 2006), for example, is also concerned with the creation of combinatorial structures, such as concepts, that might underlie human cognition. For the present purposes, however, we think SPA well-suited: it is inspired by human semantics and syntax in that its “semantic pointers” can be interpreted as perceptually grounded symbols in the sense of Barsalou (1999). SPA can also incorporate mechanisms necessary for concept grounding in terms of a rich sensorimotor experience (see Thill, 2015, for a longer discussion).

The question of when children acquire concepts can therefore be reformulated, for the present purposes, as asking at what age the corresponding semantic pointer forms. In the following, we first give a brief overview of the main computational principles in SPA (we refer the interested reader to Eliasmith, 2013, for a much more thorough discussion, including various demonstrations of cognitive and biological plausibility). We then provide the aforementioned characterization of concepts, which finally allows us to highlight directions for future work.

### 4.1. Brief overview of semantic pointers

Semantic pointers, in SPA, are vectors in a high-dimensional^5^. space. For example, the concept of a *robin* would thus be described by a vector **robin**. To specify how such a vector might be obtained, SPA takes inspiration from hierarchical structures in the human brain such as the visual cortex (Felleman and Van Essen, 1991). For example, the retinal image of a robin is successively compressed through the different layers of the hierarchy for object recognition (V1 → V2 → V4 → IT) into a representation with significantly lower dimensionality than the original retinal input. This resulting representation at the top of the hierarchy would be a semantic pointer **robVis** encoding the visual appearance of a robin.

Multiple representations can then be bound together to form a new concept. In SPA, the binding operator is *circular convolution*, denoted by ⊛, a vector operation which takes two vectors as an input and returns a vector of the same length as an output. To give an example from Eliasmith (2013), one could construct a semantic pointer for perceptual features of a robin:

$$\begin{array}{l} robinPercept=visual⊛robVis+auditory⊛robAud\\ \;+\;tactile⊛robTact+\ldots \end{array}$$

where each element in bold represents a semantic pointer. **robin** could then be defined as:

$$\begin{array}{l} robin=perceptual⊛robinPercept+isA⊛bird\\ \;+\;indicates⊛spring+\ldots \end{array}$$

There are several aspects of semantic pointers that we do not discuss here. It is, for example, possible to “read out” particular components of a semantic pointer (such as what the visual percept **RobinVis** within the overall concept of **Robin** is), and to recall the visual image(s) used in forming that particular pointer—a process that can be interpreted as a type of simulation of previous sensorimotor experience as proposed by Barsalou (Barsalou, 1999; Barsalou and Wiemer-Hastings, 2005; Barsalou, 2009). Further discussions of the underlying neural structures, necessary neural mechanisms, and biological plausibility can be found in Eliasmith (2013).

For the present purposes, it is also worth emphasizing that, although it is capable of symbolic manipulation, SPA is not a symbolic account of cognition; the semantic pointers related to any concept are not arbitrary symbols but *a compressed combination of perceptual features that make up the concept*. As such, the sensorimotor experience of a given concept by an agent plays a fundamental role in forming the concept and shaping computations that use it.

### 4.2. Characterization of richly grounded concepts

In essence, we argue throughout this paper that sensorimotor concept grounding requires a rich perspective of what the term “sensorimotor” actually entails: it is not merely sufficient to consider basic sensorimotor interaction with the external world; internal percepts (including affect, emotional components and other aspects of interoception as discussed in more detail, for example, by Stapleton, 2011) are equally important (Thill et al., 2014; Wellsby and Pexman, 2014a). We therefore postulate that the sensory features of a concept, directly perceived at a given time *t*, can be described as follows:

(1) $$\begin{array}{l} S_{t}^{D}=\sum_{i}\sum_{j}Modality_{i}^{ext}⊛\;feature_{j}\\ \;+\sum_{k}\sum_{l}Modality_{k}^{int}⊛\;feature_{l} \end{array}$$

where we omit an explicit mention of time on the RHS. Equation (1) simply captures the idea that concepts are multimodal and made up of any number of features from any number of modalities (notably, this number can also be low: constructs are not necessarily complex. In particular, a concept could consist of a single modality, for example the concept “yellow”). What matters is the direct nature of these features; by which we mean that they are not time-dependent. They could for instance relate to a color or the shape of a solid object, as acquired by the visual modality, the smoothness of a surface from a tactile modality, or an affordance elicited by a given object. They could equally relate to direct visceral feelings elicited when experiencing, for example, surprise, pleasure, or to the proprioceptive feeling of an extended arm. Affective mechanisms or emotional components (as highlighted by many, e.g., Kousta et al., 2011; Glenberg and Gallese, 2012; Newcombe et al., 2012) of concepts can be included by representing the different dimensions as internal modalities. For example, in PAD Space (Mehrabian and Russell, 1974), one might posit the following: **Pleasure** ⊛ **value_p_** + **Arousal** ⊛ **value_a_** + **Dominance** ⊛ **value_d_**.

Other sensorimotor perceptions, on the other hand, are time-dependent: movements are, for example, by definition expressed over time. We sketch such percepts as:

(2) $$S^{T}=f\left(S_{t=1,\ldots ,n}^{D}\right)$$

where the notation again chooses simplicity over being explicit since it is merely meant to be a sketch of a process that would capture temporal aspects of percepts. Here, *f*(·) is therefore a simply placeholder for a temporal function (see, for example, Pack and Bensmaia, 2015, for a discussion of neural sensitivity to temporal stimuli, and underlying computations, in both the visual and touch modalities).

We argue that Equations (1 and 2) provide a reasonable characterization of the sensorimotor experience that may ground concepts and provides a starting point for analysing concept acquisition. To address word acquisition proper, we also need to recognize that verbal labels can be attached to concepts. This gives us the first expression for a concept grounded in rich sensorimotor experience:

(3) $$C=S^{D}+S^{T}+Label⊛name$$

Next, we note that pointers in SPA can be constructed from other pointers, as in the previous example of the robin. We can introduce a similar idea here by noting that a given concept can be made up by more than just direct sensory features; it can equally include existing concepts:

(4) $$C=S^{D}+S^{T}+\sum_{i}\sum_{j}Includes_{i}⊛C_{j}+Label⊛name$$

where we highlight that other concepts are not merely added by summation (see Eliasmith, 2013); it is rather the compressed vector that is added as a property (that we refer to as **Includes** here). Equation (4) also captures how some researchers, (particularly those primarily interested in robotic models of concept grounding) believe abstract concepts can be grounded (see Stramandinoli et al., 2012, for an example and Thill et al. 2014, for a larger discussion). In such theories, rather than being grounded in direct sensorimotor features, abstract (or higher order) concepts are instead grounded in other concepts, possibly with no direct sensorimotor component at all, meaning the first two terms on the RHS of Equation (4) would be empty.

In sum, we argue that Equation (4) describes the general form of a grounded concept, can accommodate current views on concepts, can account for abstract concept acquisition, and allows us to incorporate a rich embodied experience without positing a separate mechanism. For example, the modalities that provide features can extend to the social domain, in line with claims that more abstract words go beyond the simple sensorimotor to include a stronger social component (Borghi and Cimatti, 2009, 2012; Borghi and Binkofski, 2014). It is also worth highlighting that the characterization does not require *all* components to be related to some form of sensorimotor experience (even if rich). The use of Includes allows for the inclusion of purely linguistic features (Kousta et al., 2011), which in turn allows for dis-embodied concepts in the sense of Dove (2011). Indeed, in any of the above, the left-hand term of the ⊛ operator in SPA could in principle refer to anything and does not necessarily need to be itself something that has a direct sensorimotor grounding (as is clear from the robin example above). This therefore also allows for the construction of metaphors in the sense of Lakoff and Johnson (1980)—as a crude example, one could for instance postulate the following:

(5) $$Happiness\approx Modality^{int}⊛Up$$

which is meant to express that happiness causes interoceptive feelings that are somewhat akin to the grounded concept of “Up.” **Up**, here is a concept as described by Equation (4).

Finally, it is worth pointing out that this characterization is open to the use of purely amodal symbols, perhaps even in conjunction with grounded ones. Exploring this further would require a theory of how such semantic pointers are formed, but once they are, they could be used at the appropriate places in Equations (1–4) (where one could for instance imagine a dedicated modality for amodal symbols). We do not pursue this here since our main aim is to discuss the grounding of concepts.

## 5. Discussion

Having characterized concepts in terms of the semantic pointer architecture, we now turn to ways in which it can contribute to our understanding of concept acquisition. The first thing to note is that this new account is strongly developmental. As mentioned in the introduction, concepts evolve over time—a 5 year old's concept of *love* is unlikely to be identical to that of a 15-year-old, which in turn is likely to be different from the concept the individual will have at age 35. For any given concept, its characterization in Equation (4) therefore changes over time. In particular, concepts may initially be formed from partial information and additional terms added as the modalities that provide such features develop, or other types of information becomes available, reflecting the rapid development of conceptual structures seen in early childhood (Quinn and Eimas, 1997; Mandler, 2000). The characterization given by Equation (4), for any given concept, is therefore also subject to development. Thus, it is possible to predict a developmental timeline given a hypothesis of necessary constitutents—that is, a concept can only be acquired once its constituent semantic pointers have been acquired. It is worth pointing out that any theory of concept acquisition implicitly makes at least one prediction in this sense: that the proposed cognitive mechanisms exist by the time children begin to acquire the concepts in question. As noted previously for example, Dove (2011) has argued that the ability to form metaphors develops too late to adequately be positioned at the core of abstract concept grounding (although metaphors can contribute to such concepts once available). Similarly, the idea that concepts might be made of contextualized simulations (Barsalou, 1999; Barsalou and Wiemer-Hastings, 2005; Barsalou, 2009) predicts that the necessary mechanisms to develop such simulations develops in a manner consistent with AoA. Conversely, if a developmental timeline for simulation mechanisms is given^6^, it is then possible to sketch how a concept develops from AoA onwards as the simulations it relies on mature.

A historic problem for theories of embodied cognition is how to account for acquisition of concrete and abstract concepts in a single mechanism. For example, while concrete *yellow* can be directly acquired from the external world, the more abstract *lonely* requires interoceptive features, while *whatever* is arguably linguistically mediated. Here, Equation (4) provides a starting point since it can form the basis for a measure of how much of a given concept is grounded in simple, directly perceivable sensorimotor modalities in the sense of Equations (1 and 2). In other words, how abstract a concept is is a function of how much of its substance goes beyond simple sensorimotor grounding. This is essentially very similar to the previously mentioned claims from the WAT theory (Borghi and Binkofski, 2014), which argues that more abstract concepts are made up of more social aspects that are not related to an individual's sensorimotor experience. At the same time it extends this to include *any* source for aspects that are not of a simple external sensorimotor type, including not only more complex sensorimotor experiences related to linguistic usage of the concepts (Barsalou et al., 2008; Dove, 2011; Zwaan, 2015) but also interoceptive (Thill et al., 2014) features.

Because our characterization in Equation (4) incorporates interoceptive features, the conceptual structure it entails is subtly different from that of the commonly and often interchangeably used, adult-rated concreteness or imageability scales (Reilly and Dean, 2007). By trying to provide a way to quantify how much of a concept is grounded in a rich but direct sensorimotor experience, we measure the “groundability” of a concept: the degree to which a concept is directly grounded in embodied processes. Importantly, these embodied processes include internal modalities, including affect and other interoceptive aspects: a concept can thus be directly grounded even if it has no perceivable aspect in the external world. Rather than distinguishing between “concrete” and “abstract” concepts, then, we distinguish between concepts that have a larger or smaller proportion of directly grounded components. Developing a groundability scale, in particular one that can account for development, will be key to empirical tests of this account.

The mechanisms provided by SPA also raise important questions for subsequent work: for example, since SPA uses vectors for the underlying representations, what might the distribution of these vectors be when constructed in a bio-realistic fashion, and to what degree does this relate directly to our measure of groundability? Further, a developmental process that enriches concepts over time with newly accessible information from existing or new modalities effectively modifies the direction of the vector in space. This might provide a quantitative measure for the amount of change that the introduction of a new cognitive mechanism can induce in a concept.

Importantly, this approach is also consistent with the developmental literature. Sloutsky (2010), for example, provides such an account of the neural mechanisms underlying concept learning, distinguishing between statistically “dense” and “sparse” categories (the difference being the amount of redundant information that a concept carries). Sloutsky relates these to different learning mechanisms—compression mechanisms for dense, and selection mechansisms for sparse categories. Where abstract concepts (which, in his terms are concepts that have no sensory target, such as “love”) are concerned, Sloutsky posits an important role for the executive function, and therefore PFC. Taken together, these insights combine into a developmental hypothesis of category learning: dense categories are easier to learn than sparse because the required compression mechanisms develop earlier while the involvement of the executive function in abstract concepts would predict a late acquisition due to the late maturation of the PFC (for a much more detailed reasoning, see Sloutsky, 2010). The account we have provided here includes these considerations in the precise neural mechanisms that SPA postulates to underlie semantic pointer formation (Eliasmith, 2013), but it also extends them with a more explicit inclusion of embodied mechanisms that have their own developmental timeline. Our account also ties in with Barsalou's idea of *situated conceptualization* (Barsalou, 2009) and the suggestion that concepts are a “large collection of situational representations” (Barsalou and Wiemer-Hastings, 2005, p. 156) since, as previously noted, SPA can be seen as a computational implementation of Barsalou's (1999) perceptual symbol system. A situated conceptualization could be achieved by decompressing some of the semantic pointers (thus activating simulations of the corresponding sensorimotor experience) that make up a given concept. Conversely a theory of what situated conceptualizations for a given concept need to contain can in turn provide insights into what aspects of (internal and external) sensorimotor experience might make up that concept, thus contributing to insights into the nature of Equation (4) for that concept.

## 6. Conclusion

In sum, we have shown how developmental accounts of concept acquisition can include embodied theories of cognition, without being forced to claim that all aspects of all concepts are necessarily grounded in some sensorimotor experience. We have also highlighted the importance of understanding the term “sensorimotor” experience as going beyond sensorimotor interaction with the *external* world: the inside matters just as much. We refer to the extent to which a concept is richly embodied in this way as its *groundability*. Using empirical data, we have shown both that the semantic features typically considered in developmental studies are not sufficient to explain variability in AoA and, critically, that including BOI as a measure which can be related to sensorimotor experience improves the results.

Our account unifies existing theories of embodied cognition in a single mechanism by highlighting how cognitive mechanisms that develop comparatively late can enrich existing concepts. It also makes it clear that concepts which have no components that are available early on can only develop later. It also suggests that additional factors in AoA cover a range of attributes: (a) the complexity of the underlying concepts in terms of how many modalities and features they aggregate, (b) the proportion of directly groundable features, (c) the degree to which such features refer to aspects of the external sensorimotor experience, (d) the development of necessary sophisticated mechanisms, and (e) the ability to communicate about them. Thus, this theoretical account integrates research in embodied cognition and cognitive development, paving, we hope, the way for future empirical tests of the interaction between groundability and concept acquisition.

## Author contributions

All authors listed have made a substantial, direct and intellectual contribution to the work, and approved it for publication.

### Conflict of interest statement

The authors declare that the research was conducted in the absence of any commercial or financial relationships that could be construed as a potential conflict of interest.

## Footnotes

^1^In contrast, non-representationalist theories of embodied cognition are an evolution of Ecological Psychology and its precursors.

^2^Stapleton (2013) actually omits the “may,” stating that “I argue that recent work in neuroscience and robotics suggests cognitive systems are not merely superficially embodied in the sense that the sensorimotor interactions with the environment are the only interactions relevant to cognitive behavior, but that cognitive systems are ‘properly embodied’; the internal body matters to cognition” (pp. 1–2).

^3^Details available at http://www.psych.rl.ac.uk/MRC_Psych_Db_files/mrc2.html.

^4^In PAD space (Mehrabian and Russell, 1974), for example, valence ratings do not relate to the A or the D.

^5^Eliasmith (2013) suggests that 500 dimensions are sufficient for human cognition.

^6^Thill and Svensson (2011) discuss the current lack of such a timeline in more detail and speculate that simulations may co-develop with dreams, with the implication being that the quality of dreams (which do not reach adult-levels of sophistication until the late teens) may serve as an indicator of the sophistication of internal models underlying simulations.
