# Supplementary material for: What's on the Inside Counts: A Grounded Account of Concept Acquisition and Development
Source: Front Psychol. 2016 Mar 23;7:402. doi: 10.3389/fpsyg.2016.00402 (PMC4804724; doi:10.3389/fpsyg.2016.00402)
Supplement: Supplementary file 1 [file List_of_data.pdf]

List of data used in the regression analyses of Thill & Twomey (2016; doi: 10.3389/fpsyg.2016.00402)

Word	AoA (months)	CHILDES Frequency	Imageability	Valence	BOI
animal	23	396	575	6.48	
ankle	30	21	613	5.27	
apple	17	973	637	6.41	5.26
arm	21	354	593	5.34	5.7
aunt	24	196	567	6.39	4.73
baby	15	6227	608	8.22	5.59
bad	23	634	388	2.56	
banana	16	536	644	6.61	4.89
basement	31	77	571	4.67	3.29
basket	25	410	560	5.45	5.07
bath	17	492	601	7.33	4.8
beach	23	377	667	8.03	4.3
bear	19	2201	572	4.78	
bed	20	1663	635	7.51	6.27
bench	23	54	555	4.61	5.9
bib	20	216	488	5.57	3.9
bird	16	1128	614	7.27	5.17
black	29	665	589	5.39	
blanket	19	262	582	6.94	5.78
blue	22	2068	569	6.76	1.55
boat	18	389	631	7.79	5.7
book	16	4936	591	5.72	6.33
bottle	16	718	619	6.15	5.59
bowl	21	603	579	5.33	5.93
boy	20	3383	618	6.32	5.67
break	23	647	398	4.59	
broken	22	410	469	3.05	
broom	21	109	608	4.83	6.31
brother	28	242	589	7.11	
bucket	26	252	586	5.1	5.11
bunny	19	1379	585	7.24	
butter	22	421	603	5.33	4
butterfly	23	302	624	7.17	2.52
button	20	474	580	5.21	4.96
cake	22	519	624	7.26	5.9
candy	22	266	601	6.54	
car	25	2529	638	7.73	6.4
cereal	22	439	576	7.35	
chair	19	2016	610	5.08	
chalk	30	58	601	4.89	5.6
cheese	18	986	592	6.33	
chicken	22	749	619	6.87	3.67
child	28	153	619	7.08	6.07
chin	22	124	608	5.29	4.4
chocolate	19	438	611	6.88	5.49
church	26	58	616	6.28	4.36
circus	31	155	586	7.3	
clean	23	1361	454	7.23	
clock	20	208	614	5.14	5.47
closet	26	174	525	5.21	2.96

Word	AoA (months)	CHILDES Frequency	Imageability	Valence	BOI
clown	23	310	589	5.39	4.67
cold	19	992	531	4.02	
cook	23	309	504	6.16	
cookie	16	1300	600	7.6	5.15
corn	23	276	601	6	5.93
couch	23	272	536	6.78	5.86
country	31	64	539	5.93	
cow	19	848	632	5.57	
cup	19	1033	558	5.44	5.79
cut	26	701	460	3.64	
dance	22	349	510	7.38	
dark	25	274	586	4.71	
day	29	1793	526	6.66	1.87
dinner	22	627	570	7.16	
dirty	19	876	485	3.08	
doctor	23	357	600	5.2	3.37
dog	14	1385	636	7.57	6.4
doll	20	315	565	6.09	6.43
door	19	1250	599	5.13	
dress	23	366	595	6.41	
dump	30	289	528	3.21	
eat	19	5076	563	7.47	
egg	21	674	599	5.29	
elephant	31	593	616	6.48	1.93
eye	16	543	603	5.86	5.47
face	23	1299	581	6.39	5.8
fall	22	933	547	4.09	
farm	27	346	560	5.53	4.1
finish	29	830	437	7.8	
first	27	1927	388	6.89	1.27
fish	21	1175	615	6.04	5.73
flag	26	51	607	6.02	
flower	19	424	618	6.64	4.33
food	23	1149	539	7.65	6.4
foot	19	899	597	5.02	5.73
fork	21	205	598	5.29	6.13
friend	27	591	587	7.74	5.53
frog	31	417	617	5.71	5.03
game	27	664	521	6.98	3.97
garbage	23	278	596	2.98	4.07
garden	30	191	635	6.71	5.22
gentle	30	268	422	7.31	
girl	22	2264	634	6.87	5.13
give	22	3477	383	7.13	
glass	23	255	585	4.75	5.83
good	22	11108	374	7.47	
grass	22	293	602	6.12	5.3
green	25	1869	609	6.18	1.43
hair	19	1515	580	5.56	5.8
hammer	23	284	618	4.88	5.37
hand	19	1607	598	5.95	5.87
happy	23	983	511	8.21	
hard	28	1049	460	5.22	
hat	18	1479	562	5.46	6.07
hate	31	58	462	2.12	

Word	AoA (months)	CHILDES Frequency	Imageability	Valence	BOI
head	21	1719	593	6.63	6.03
heavy	23	335	495	3.69	
hen	30	85	597	5.1	4.31
hide	25	312	430	4.32	
high	27	469	463	6.62	
home	22	1770	599	7.91	4.23
horse	19	646	624	5.89	
hose	25	60	572	5.25	4.47
house	22	2458	606	7.26	
hungry	23	706	503	3.58	
hurt	24	922	465	1.9	
ice	22	413	635	5.92	5.79
jar	30	109	571	5.21	
jelly	25	134	590	5.66	
juice	16	1845	593	6.79	5.9
kick	23	205	551	4.31	
kiss	21	896	633	8.26	
knee	21	131	597	5.03	5.17
knife	30	145	633	3.62	6.07
lamb	26	181	614	5.89	5
lamp	28	56	575	5.41	5.48
leg	22	385	601	5.71	5.96
like	25	17537	352	7.52	
lion	23	412	626	5.57	1.93
listen	29	504	378	5.93	
loud	27	317	448	4.77	
love	23	1434	569	8.72	2
lunch	24	751	602	7.21	4.8
mad	29	293	479	2.44	
man	22	1161	567	6.73	6.3
me	20	14537	430	8.06	
meat	24	250	618	6.66	6
medicine	23	200	551	5.67	4.8
milk	19	1346	638	5.95	5.3
money	22	748	604	7.59	5.1
moon	21	512	585	6.74	2.33
mouth	19	1790	613	5.46	
movie	29	316	571	6.86	3.1
nail	27	72	588	5.14	5.97
napkin	23	248	582	4.84	5.39
necklace	24	95	606	6.39	5.19
nice	25	3259	375	6.55	
night	23	1114	607	6.06	1.53
noisy	28	133	215	5.02	
nose	16	1674	605	4.71	5.43
nurse	31	195	617	6.08	5.3
old	30	977	478	3.31	
orange	22	1114	626	6.47	5.15
oven	27	157	599	5.71	4.78
owl	22	258	595	5.8	4.17
paint	26	263	567	5.62	5.3
paper	21	1354	590	5.2	5.93
party	25	422	596	7.86	4.39
pencil	25	297	607	5.22	5.96
penny	25	85	609	5.06	
people	26	1404	548	7.33	
person	31	338	562	6.32	

Word	AoA (months)	CHILDES Frequency	Imageability	Valence	BOI
pig	19	670	635	5.07	5.23
pillow	21	215	624	7.92	5.78
plant	25	198	605	5.98	5.63
plate	23	351	527	5.3	5.5
play	23	5885	498	8.1	
pony	26	106	642	6	
pool	20	200	577	7.7	5.37
poor	31	468	447	2.28	
porch	31	37	586	6.14	4.57
present	23	281	481	6.95	3.93
present	23	281	481	6.95	3.93
pretty	22	2185	520	7.75	
puppy	19	693	635	7.56	
quiet	25	295	426	5.58	
radio	26	86	613	6.73	4.04
rain	20	322	618	5.08	4.27
red	23	2097	585	6.41	1.61
refrigerator	25	221	612	6.14	4.48
rock	21	360	612	5.56	
roof	30	101	604	5.4	3.14
room	23	1548	545	5.52	4.93
sad	27	399	419	1.61	
salt	22	101	570	5.56	5.4
school	23	1550	599	4.36	4.69
scissors	25	143	609	5.05	5.48
sheep	23	438	596	6.44	5.31
shower	22	102	615	7.04	4.33
sick	26	316	456	1.9	
sing	25	953	527	6.77	
sister	29	270	613	7.46	
skate	31	28	563	6.6	4.1
sky	23	361	618	7.37	1.53
sleep	22	863	530	7.2	3.1
slow	30	213	377	3.93	
smile	27	123	615	8.16	2.73
snow	23	650	597	7.08	
soap	20	193	600	5.97	6.27
sofa	31	49	597	6.53	5.27
soft	26	300	476	7.12	
soup	23	342	604	6.25	5.7
spoon	19	784	584	5.93	5.97
star	24	390	623	7.27	2.23
stop	24	1600	452	3.96	
store	22	801	506	5.93	4.23
story	23	1205	491	6.63	2.56
stove	26	123	592	4.98	
street	25	348	577	5.22	4.2
sun	23	569	639	7.55	2.13
table	23	1391	582	5.22	5.04
taste	29	397	425	6.66	
teacher	29	189	575	5.68	
think	31	10902	384	6.41	

Word	AoA (months)	CHILDES Frequency	Imageability	Valence	BOI
thirsty	25	236	482	3.61	
tickle	22	412	492	6.86	4.19
tiger	23	240	606	5.89	1.67
time	31	3382	413	5.31	2.03
tired	25	751	419	3.28	
tooth	19	169	624	5.19	5.9
touch	26	912	456	6.31	
towel	22	380	570	5.75	6.22
toy	19	885	569	7	6.17
train	20	1120	593	5.59	5.14
trash	29	196	599	2.67	5.2
tray	31	179	550	5.1	5.29
tree	19	1011	622	6.32	5.53
truck	18	1239	621	5.47	
turtle	23	308	564	6.78	2.93
watch	25	1789	525	5.78	
water	19	2570	632	6.61	
wet	21	597	509	5.57	
white	27	873	566	6.47	1.5
window	23	568	602	5.91	3.52
wish	31	177	399	7.09	1.87
wolf	27	116	610	5	4.7
work	23	1266	458	3.96	2.7
yellow	25	1429	598	5.61	
zipper	22	162	632	5.39	5.04
